# Refuting phylogenetic relationships

**DOI:** 10.1186/1745-6150-1-26

**Published:** 2006-09-06

**Authors:** James Bucknam, Yan Boucher, Eric Bapteste

**Affiliations:** 1 Canadian Institute for Advanced Research and Genome Atlantic, Department of Biochemistry and Molecular Biology, Dalhousie University, Halifax, Nova Scotia, B3H 4H7, Canada; 2 Department of Chemistry and Molecular Biosciences, Macquarie University, North Ryde, NSW 2109, Australia

## Abstract

**Background:**

Phylogenetic methods are philosophically grounded, and so can be philosophically biased in ways that limit explanatory power. This constitutes an important methodologic dimension not often taken into account. Here we address this dimension in the context of concatenation approaches to phylogeny.

**Results:**

We discuss some of the limits of a methodology restricted to verificationism, the philosophy on which gene concatenation practices generally rely. As an alternative, we describe a software which identifies and focuses on impossible or refuted relationships, through a simple analysis of bootstrap bipartitions, followed by multivariate statistical analyses. We show how refuting phylogenetic relationships could in principle facilitate systematics. We also apply our method to the study of two complex phylogenies: the phylogeny of the archaea and the phylogeny of the core of genes shared by all life forms. While many groups are rejected, our results left open a possible proximity of *N. equitans *and the Methanopyrales, of the Archaea and the Cyanobacteria, and as well the possible grouping of the Methanobacteriales/Methanoccocales and Thermosplasmatales, of the Spirochaetes and the Actinobacteria and of the Proteobacteria and firmicutes.

**Conclusion:**

It is sometimes easier (and preferable) to decide which species do not group together than which ones do. When possible topologies are limited, identifying local relationships that are rejected may be a useful alternative to classical concatenation approaches aiming to find a globally resolved tree on the basis of weak phylogenetic markers.

**Reviewers:**

This article was reviewed by Mark Ragan, Eugene V Koonin and J Peter Gogarten.

## Open peer review

Reviewed by Mark Ragan, Eugene V Koonin and J Peter Gogarten. For the full reviews, please go to the Reviewers' comments section.

## Background

Since the 1960's, molecular phylogeneticists have sought to reconstruct organismal relationships based on gene and protein trees [1]. Generally, successes in this enterprise have been evaluated as a function of the capacity to build unambiguous monophyletic groups, thus reducing the paraphyly of former classifications [2]. Despite such precise goals, tree reconstruction remains notoriously difficult, both for practical and conceptual reasons. Regarding some of the problems of classical phylogenetics, many publications have presented what we feel to be valid arguments [3]. At a large scale, the most pervasive issue is probably the occurrence of lateral gene transfers (LGT) [4]. This paper will, however, focus on a different topic, tackling instead a peculiar practical issue: the relative weakness and the ambiguity of the phylogenetic signal in a context of a tree-like pattern of evolution.

Dealing with phylogenetic information recorded in molecular markers is indeed often a major challenge. Each individual gene is of limited size, generally too short to fully resolve a given phylogenetic question [5]. In addition, the phylogenetic signal is generally eroded by mutational saturation and suffers from other sources of homoplasy such as convergence or parallelism that led to unevenly resolved trees. This unequal support is reflected by the bootstrap values (BVs) associated with the different nodes of the gene tree. BVs are the most common way to assess the robustness of clades. They are produced by a random resampling of positions with replacement to determine sampling error or the confidence interval for the groups as displayed in the hypothesized best tree [6]. Low BVs reflect that the marker employed contained too few synapomorphies to be conclusive. High BVs indicate that the data consistently support a given grouping. However to conclude that a highly supported grouping identifies a true set of descendants from a common ancestor requires that the BV statistics are not artefactually biased. Such artifactual biases often arise, since very fast-evolving species of an alignment will systematically tend to group together, independently of their real phylogenetic relationships [3]. If present, this ***long branch attraction ***problem is generally aggravated as the sequences under study get longer [7]. Such artifically increased BVs are also observed in self-concatenation (i.e. concatenation of a single marker with itself). While no new independent data are added, the simple repetition of the same characters leads to questionnable higher supports (data not shown).

In spite of this, when phylogeneticists face problems of weak resolution using a few markers, they traditionally try to increase the support for the clades under study by providing more phylogenetic signal (i.e. increasing the sequence length) [8]. That is, they study simultaneously several genes or proteins in an attempt to raise the global quantity of synapomorphies available to support each monophyletic group, following a principle of ***total evidence***. As one often reads in papers proposing concatenation analyses, total evidence rests on the assumption that if multiple markers share a common history then their overall analysis will produce a concatenation tree that is better resolved, through summation of their individually weak phylogenetic signals.

As debates around the works of the famous philosopher R. Carnap have shown, total evidence methods have a defined epistemological background: they belong to the ***verificationist ***toolkit. In other words, aware of it or not, classical phylogeny and its attempt to establish robust clades by concatenation are a verificationist procedure. Importantly, verificationism is not the only possible approach to acquiring scientific knowledge, as decades of epistemological studies have underlined. For instance, it is well known that falsificationism directly opposes verificationism. Falsificationism supposes that "*natural laws ... can never do more than exclude certain possibilities*"[9]. To make the constrast more clear in a phylogenetic context: instead of aiming to confirm clades *via *a progressive increase in their bootstrap support when more data is added, a falsificationist would more likely be interested first by the rejection of impossible groupings, underlining what **can not be a natural group**. These divergent goals introduce some asymetry in our way of looking at phylogenetic results. We will argue that it can sometimes be easier (and preferable) to decide which species do not group together than which ones do. Our reason to encourage the development of an alternative perspective to verificationism is not a commitment to falsificationist *sensu stricto*. Simply, it follows a third epistemological current: the one of P. Feyerabend, who stated that: «*proliferation of theories is beneficial for science, while uniformity impairs its critical power*» [10].

In this case, we feel that more falsificationist perspectives should be explored as a counterbalance to the current verificationist foundation of molecular phylogenetics. We illustrate this by describing a very simple tool whose objectives are to refute impossible phylogenetic relationships, highlighting disagreements and inconsistencies about *a priori *possible clades, not exclusively their consistency. In this way, instead of reaching a weak conclusion – we cannot know for sure if a given clade A exists – we can make the strong claim that another group B is certainly not an appropriate unit in any natural classification. Typically, our «falsificationist-lite»-software could be useful for those who study a new genome and want to test rapidly and without taxonomic *a priori *assumptions, which relationships are rejected by their multiples alignments, that is, to which taxa the genome could never belong. Our software also provides the details of the pattern of rejection for each gene, so that markers supporting atypical impossible groupings can be easily identified, suggesting specific instances of phylogenetic artefact, lateral gene transfer or hidden paralogy for these markers. Finally, through its identification of impossible associations, our method delineates restricted sets of possible relationships that could help traditional (verificationist) classifiers explore a refined selection of realistic taxonomic connections.

## Material and methods

### Constitution of the datasets

The archaeal dataset is the one presented in [11]. In brief, it comprises 23 aligned ribosomal markers: rpl2p, rpl15p, rpl18p, rpl22p, rpl23p, rpl30p, rpl37ae, rpl3p, rpl44e, rpl4p, rps10p, rps13, rps15p, rps17e, rps19e, rps19p, rps2p, rps3p, rps4p, rps5p, rps6e, rps7p and rps8e. Each marker presented 23 species: 4 crenarchaeotes (*Pyrobaculum aerophilum*, *Aeropyrum pernix*, *Sulfolobus solfataricus*, *Sulfolobus tokodaii*), 3 Thermococcales (*Pyrococcus furiosus*, *Pyrococcus abyssi*, *Pyrococcus horikoshii*), 3 Thermoplasmatales (*Ferroplasma acidarmanus*, *Thermoplasma volcanium*, *Thermoplasma acidophilum*), 2 Halobacteriales (*Halobacterium sp*, *Haloarcula marismortui*), 4 Methanosarcinales (*Methanosarcina barkeri*, *Methanosarcina mazei*, *Methanosarcina acetivorans*, *Methanoccoides burtonii*), 1 Methanobacteriales, (*Methanothermobacter thermoautotrophicus), 2 Methanococcales (Methanocaldococcus jannaschii, Methanococcus maripaludis) Archaeoglobus fulgidus*, diverse methanogens (*Methanogenium frigidum Methanopyrus kandleri*) and *Nanoarchaeum equitans*.

The sequences of 34 genes identified as core genes in [12] were retrieved using the program "retrieve sequences" in all analyses of the Neurogadgets website [38] (option "Reciprocal best match in other genomes" using a GI number). The 34 genes were: *argS*, *infB*, *pheS*, *rplN*, *secY*, *dnaG*, *ksgA*, *proS*, *rpsB*, *serS*, *dnaX*, *leuS*, *rplA*, *rpsC*, *thrS*, *fusA*, *lysS*, *rplC*, *rpsD*, *trpS*, *gcp*, *metG*, *rplE*, *rpsG*, *valS*, *gltX*, *nusA*, *rplF*, *rpsH*, *ychF*, *hisS*, *nusG*, *rplK*, *rpsM*. For all these markers, we produced a careful alignment and preliminary phylogenetic analyses (NJ) to check the sequence orthology. We subsequently excluded from the files all the instances of species harboring multiple copies of each gene and obtained a set of 34 files with 135 shared species. Maximum Likelihood analyses, using Phyml [13], were conducted on these data to ensure that the monophyly of the main groups under study was supported. As it appeared that the homology of archaeal and bacterial sequences in *lysS *was doubtful, and that *pheS *and *proS *presented either hidden paralogy problems or more likely ancient LGTs between the two prokaryotic domains, these 3 markers were removed for the rest of our study. We used a selection of 43 common species representative of 8 major prokaryotic groups in the 31 remaining markers, for further in-depth phylogenetic analyses to be presented here and elsewhere. The groups tested here were: the Archaea (*Halobacterium sp., Pyrococcus abyssi*, *Archaeoglobus fulgidus*, *Methanosarcina acetivorans*, *Thermoplasma volcanium*, *Pyrobaculum aerophilum, Aeropyrum pernix, Sulfolobus solfataricus*), the Spirochaetes (*Borrelia burgdorferi*, *Treponema denticola*, *Treponema pallidum*), the Chlamydiales (*Parachlamydia sp*., *Chlamydia muridarum*, *Chlamydia trachomatis*, *Chlamydophila pneumoniae*, *Chlamydophila caviae*), the actinobacteria (*Propionibacterium acnes*, *Bifidobacterium longum*, *Mycobacterium avium subsp paratuberculosis*, *Corynebacterium diphtheriae gravis*), the Proteobacteria (*Campylobacter jejuni*, *Wolinella succinogenes*, *Desulfovibrio vulgaris*, *Geobacter sulfurreducens*, *Xanthomonas axonopodis*, *Nitrosomonas sp*., *Caulobacter crescentus*, *Mesorhizobium loti*), the Cyanobacteria (*Gloeobacter violaceus*, *Synechocystis sp*., *Prochlorococcus marinus*, *Thermosynechococcus elongatus*, *Nostoc sp*., *Synechocystis sp*.), the Aquificales/Thermotogales (*Thermotoga maritima*, *Aquifex aeolicus*) and the firmicutes (*Clostridium acetobutylicum*, *Thermoanaerobacter tengcongensis*, *Ureaplasma parvum*, *Mesoplasma florum*, *Lactobacillus johnsonii*, *Bacillus subtilis*, *Staphylococcus aureus*).

### Impossible analyses

Bootstrap calculations were calculated using PHYML [13] (settings, 4 categories, Γ law, JTT model of evolution) and CONSENSE [14] for each of the 31 markers. These bipartitions were inputed to our new software: Impossible, which accepts as input any bipartition files produced by CONSENSE [14], containing identical or overlapping set of species. Missing taxa are not a problem, as an option allows us to filter the outputs to focus on the common species, if desired. Typically, these infiles, one per marker, contain the list of the species investigated and the list of bipartitions with their BVs. Bipartitions appear as a string of "*" and ".", which splits the species investigated into two disjoint subsets. Species sharing a "*" or species sharing a "." are more closely related than species with a different symbol. In classical phylogeny, each bipartition, thus defining a split, allows us to draw a dichotomy on a tree, and the bootstrap support indicates the robustness of this relationship. Impossible examines all of the bipartitions that have support above a user-defined cutoff to determine two different types of impossible relationships: the basic impossibility and the combined impossibility (see Additional file 1).

Basic impossibilities occur between the two subsets of a single bipartition (i.e. AB|CDE or **...): grouping members of the left subset with members of the right subset is impossible, and vice versa. By default, basic impossibilities are given a score of one. The higher the score between two species or two groups of species, the more their association is rejected by the data. Combined impossibilities are quite different from basic impossibilities as they explore the hierarchical structure of the phylogenetic tree, and involve more than one bipartition. Each combined-impossibility consists of two subsets of species which come from n different bipartitions and have disjoint taxonomical sampling. To identify them, for each marker, all pairs of bipartitions are examined to determine if a combined impossibility exists between them. For instance, for the bipartitions ABC|DEF and AB|CDEF, there are four pairs of cross-subsets: ABC-AB, ABC-CDEF, AB-DEF, and CDEF-DEF. However, in our definition, only AB-DEF is a combined-impossibility since all the other pairs, sharing species between their subsets, are partly overlapping. Each combined impossibility is given a score of one plus their degree value. Conceptually, this degree value represents the number of strongly supported nodes which occur on the path between the two subsets of the cross impossibility and thus oppose to their direct grouping. To calculate this degree value, we defined a 'related set' as any set that includes one member of the combined-impossibility as a subset. We then listed the related sets present in all the bipartitions of a marker for each subset of the combined-impossibility of interest. Our second step is to evaluate the number of related sets that do not share any species in common. Indeed, the list of related sets does not correspond exactly to the number of dichotomies between two combined-impossible subsets of species as some of the related sets partially overlap and encompass common species. In order to detect and remove the overlaps between 'related sets' (not to obtain artefactually high degree values of impossibilities) and to define the number of nodes separating two groups, we sort them by decreasing order of size, based on the number of species that they encompass.

Briefly, 'related sets' with the most species are more likely to be involved in an overlap. The related sets that are both involved in the most numerous overlaps and higher in our list are then removed. In case of ties, one of the related sets involved in the largest number of overlaps is randomly selected for removal. The procedure is repeated until no overlap is left. By default, the degree of this combined-impossibility is then evaluated as the number of remaining related sets plus one (see Additional file 2). Researchers are however able to change these weightings in order to give cross impossibilities more or less emphasis. If the user specifies «linear» weighting, the weighting becomes the number of related sets + 1 + a user-supplied value, if he specifies «multiplicative» weighting, the weighting becomes the number of related sets + 1 multiplied by a user-supplied value, if he specifies «exponential» weighting, the weighting becomes the number of related sets + 2 exponential a user-supplied value.

Finally, these scores of impossibilities are used to compile the tables and diagrams outputted by Impossible (see Additional file 1). The tools propose 4 different summaries: (1) an impossibility diagram, (2) a group-group diagram, (3) a species-group diagram, and (4) a pairwise impossibility diagram. Each of these diagrams, while based on the same data, displays a different facet of the results. The first set of diagrams displays all of the impossibilities that the program has identified. This set contains three different sections: (a) basic impossibilities, (b) combined impossibilities, and (c) a summation of basic and cross impossibilities. On (1), the impossibilities are displayed as coloured squares sets on a grid. Each column corresponds to a different markers, whereas each row corresponds to an individual impossible association of species, supported by one marker under investigation. Therefore, a square in column X in the row corresponding to impossibility w-y indicates that the association of w with y is impossible for the marker X. One can thus assess which markers present similar pattern of refutation and which do not, and which relationships are strongly rejected, or only rejected by a few markers. To ease this identification further, the rows are ordered to locate the impossibility that occurs in the largest number of markers at the bottom of the diagram, while the rarest impossible relationships is placed at the top of the diagram. In both (1a) and (1b), basic impossibility squares are coloured in white, and combined-impossibilities are indicated with various shades of blue, with darker shades corresponding to a higher overall degree value. The numerical values indicating the strength of the rejection of a relationship are also displayed in an array, for further statistics (outfile .pharm). Finally, in the combined diagram (1c), an extra-row and an extra-column display the overall impossibility score for that whole row or whole column. Markers proposing the most impossibilities (refuting genes), and strongest impossibilities (refuted groups) receive the highest overall score, and are easily identified.

The second diagram, which is known as the group-group impossibility diagram, transforms the data included in the first diagram in a bar graph. The length of each bar represents the total score for a particular impossible grouping. For instance, the entry AB would show the sum of the values for all of the impossibilities that have AB as a subset. Diagram (2) thus allows a researcher to find which groups of species tend to be the most unrelated to other species, and thus would deserve further investigations to be classified. This diagram presents another interesting property: the groups with the highest impossibility scores relative to other groups are also the most probable associations of species.

The third kind of diagram breaks down the information found in the group-group diagram, as it provides the users with the possibility to highlight the impossible relationships for a species of interest (i.e. their favorite bug or newly sequenced organisms). Any selected species can be given its own diagram of type (3)-the species-group diagram – that summarizes its non-relation with other groups. The species-group diagram can be used to determine in which groups the selected species cannot be placed. Counter-instances in a given marker presenting the grouping of the species with its usually refuted partners, would thus help to unravel interesting exceptions and original gene histories (i.e. LGT).

The final diagram produced by Impossible is the pairwise impossibility diagram (4). It shows impossibilities between pairs of individual species. By only showing pair relationships, this diagram avoids any possible group size bias, which tend otherwise to award large groups high impossibility scores. This is because large groups contain more elements which can be involved in impossibility relations. By using only single species, there is always only a one-to-one relationship. This diagram is also converted into numbers that can be used in statistical analyses to identify species with similar/different patterns of refutations of relationships.

Finally, Impossible contains a rough feature to test the inconsistency of *a priori *user-defined original groupings, switch -g. Although a researcher can manually look through the complete diagrams to determine which groups are refuted and which ones are not, a more user-friendly method might be to input predefined groups of interest into Impossible (i.e. the cabozoa) and have Impossible return their information. As the user-defined group to be tested will appear in the pairwise impossibility diagram under the appelation chosen by the user, an entry showing the internal impossibilities between the members of the predefined group will also be indicated in the pairwise diagram. In order for a group to be considered potentially solid, a rough estimate is that the sum of its external impossibilities (the number of associations rejected by a group) must be greater than the sum of its internal impossibilities. Otherwise, the members of the *a priori *groups will reject their grouping more than they reject their association to any other taxa.

For the archaeal dataset, we ran Impossible with the following command-line:

Java Impossible F1 0.9 -a -g F2 -o F3, with F1 corresponding to the list of files of bipartitions, F2 being the description of groups to be tested by the rough estimate of inconsistency and to be listed in the pairwise diagram and F3, the name of the outfile at the pdf format. As test-groups for F2, we retained the Methanococcales/Methanobacteriales, the Methanosarcinales, the Crenarchaeotes, the Halobacteriales, the Thermococcales, the Thermoplasmatales as described above. F2 exact format is built as the name of the group followed by the names of the species of the group, separated by a coma (i.e. groupName1 species1, species2, species3 and, on the next line, groupName2 species2, species3, species4.) 0.9 indicates that all the bipartitions with more than 10 % of support were considered. For the prokaryotic core dataset, we first ran Impossible with the following command-line: Java Impossible F1 0.1 -a -o F3, respecting the same nomenclature than above in the text. 0.1 means that biparitions with a support higher than 1-0.1, which is 90%, were considered.

The principal component analyses were run using R and a script presented in [15]. The coordinates of the species of the principal component analyses were extracted and used to test the normality of the data as well as the two-sample T-test and the Wilcoxson rank-sum with NCSS Junior 6.0. This program, which is a freely-distributed and can be downloaded from the NCSS website [39], runs on any version of Windows. The data are inputted into the program in the form of a tab-delimited table (although space-delimited and comma-delimited tables are also acceptable). This table can be prepared using our script MakeGroup, with the command line MakeGroup group pgroup, where group is a text file containing the group to be tested, as described above for Impossible and pgroup is simply the output of the Principal Component Analysis, presented on two columns. Each column represents a list of values to test different species grouping (i.e. the X axis or Y axis coordinates on the principal component analysis). To run the analysis (options Run > Run Analysis), the columns of data of interest have to be selected in the Response variables section of the options Analysis > T-tests > Two-sample t test. The output from the program will be displayed on the screen. In addition to the primary statistical tests mentioned above, NCSS also runs several other tests to determine the validity of the data such as tests of the normality and variance equality. If the normality test fail for a certain pair of groups, as its variance tests determine that the sample groups have unequal variances, it is still possible to use the Aspin-Welch Unequal-Variance test instead of the two-sample T-test to assess if two groups of species are statistically different or not. It was not needed here.

## Results and discussion

### Toward an attempt to facilitate classical molecular systematics

Classical molecular systematics embraces a tree-like model: its ultimate goal is the elaboration of a unique inclusive hierarchy [16]. Whenever a new species is discovered and its genes or genomes sequenced, the affiliation of this species to known groups is the object of the systematicist's speculations. To achieve this, significant reorganizations of former classifications may be required and some past hypotheses challenged. In such cases, multiple taxonomical levels have to be redefined or even invented. Thus the state of classification becomes then temporarily open. As with any new problem, one of the simplest positions is to assume that everything is possible, that many groups might potentially host the new species.

T. Cavalier-Smith's writings about the «cabozoan hypothesis», (the claim that there is a common origin for euglenoid and chlorarachnean chloroplasts), illustrate this logic of *a priori *maximal possibility well. As this author underlined in a paper from 1999 [17], "*a relationship between euglenoid and chlorarachnean chloroplasts has not been previously suspected apart from one brief mention of the possibility [...] Though at first sight a strikingly different arrangement, it does not actually preclude a direct phylogenetic sister relationship between them, as postulated here*". T. Cavalier-Smith used the absence of evidence as a starting point for his revised systematics. For example, he argued that for "cabozoan", "*the phylogeny of both groups is imperfectly known. [...] But this is no serious objection to the cabozoan theory*", for discicristates, that "*there is no particular reason to think that archezoa/discicristates are sisters to all eukaryotes except Reclinomonas. So I suggest that they may instead be sister only of the Cercozoa*", for the phylogenetic proximity between the sporozoan and the dinoflagellates plastids, he recalled that "*the published [tufA] trees do not rule out a direct relationship instead with dinoflagellates, for which no tufA sequences are yet available*" [17]. Open exploration of the taxonomically possible is definitely an important part of the traditional classification task.

Yet, discussing the possibility of new groupings is only the first step in such an analysis. Once a group and its subsequent sister-groups are proposed, these hypotheses must be tested. In pursuing the cabozoan case, Cavalier-Smith made it clear that "*one way of testing the cabozoan theory is to establish which of the clades postulated here are correct and which are false*". The test may not be easy, due to the weakness of phylogenetic markers [18], which is often the first problem with testing taxonomic hypotheses. Along this line of thought, Cavalier-Smith recognized that "*our inability to infer the correct branching order of the seven taxa *[including sporozoan, dinoflagellates, euglenoids and chlorarachnean] *merely from rRNA trees cannot be seriously questioned. Therefore such trees cannot be used to confirm or refute my postulates of plant, chromist, and chromalveolate monophyly*" [17]. Better resolution (at least locally) is thus often needed to refute some groupings. Yet obtaining better apparent resolution is not sufficient. Some visibly robust results on phylogenetic trees should be interpreted with caution due to potential artefacts that would unite some unrelated species in an erroneously supported single group. Another of T. Cavalier-Smith's proposed clades, the myosozoans, illustrated this issue and how the systematist should not take a phylogeny at face value without a critical look. Some results may simply be «too nice» to be considered true, i.e. "*the extreme divergence of the dinoflagellate sequences might be expected to cause them to group with the sporozoan sequences even if they were not directly related*" [17]. In this case, the comparison with other gene phylogenies, especially those including species with slower evolving genes [19], might help to pinpoint gene-specific artefactual groupings. The contrast of multiple independent gene phylogenies is thus another key in testing taxonomic hypotheses.

Consequently, these few examples stress the value in systematics of developing phylogenetic approaches aimed at testing multiple relationships, possible and impossible alike: accounting both for the limited as well as for the conflictual resolutions between multiple gene trees.

### Epistemological principles of our tests of phylogenetic hypotheses

With this goal in mind, we developed phylogenetic analyses testing hypothetical groupings but trying to be less verificationist and more falsificationist in spirit. To make this last principle explicit, we will discuss in more detail the interests and scopes of these two different epistemogical approaches.

The notion that cladism and total evidence are verificationist is indeed often argued in the literature [20]. For instance, «*introducing the concept of 'total evidence' in systematics, Kluge (1989) cited the relevant theoretical background, namely the work on inductive inference and its relevance for epistemology by the philosophers Rudolf Carnap and Carl Hempel. Consulting the references provided by Kluge (1989) of Carnap (1950) and Hempel (1965) shows that for these authors, the principle of 'total evidence' was tied to inductive inference*» [21]. More precisely, «*the empiricist philosopher Rudolf Carnap used 'total evidence' as a tool of decision-making, where the decision is to accept or reject a certain theory/hypothesis on inductive grounds. 'Total evidence' supports this process of decision-making by determining, in part, the value of a 'c-function,' which is the 'degree of confirmation'*» [22]. «*If [evidence] e expresses the total knowledge of X at time t, that is to say, his total knowledge of the results of his observations, then X is justified at this time to believe [hypothesis] h to the degree r [where r is the result of applying inductive logic to e and h], and hence to bet on h with a betting quotient not higher than r.]*" [23]. Put in more concrete phylogenetic terms, total evidence suggests to analysing as many data as possible to decide how much support a given relationship has. In concatenation analyses, this is achieved by joining all the genes together in the largest alignment possible, thus maximising the number of synapomorphies.

The logic of verificationism developed here has consequently one limit: such an «*inductive support works symmetrically, confirming or disconfirming theories or hypotheses to a greater or lesser degree. An empirically confirmed hypothesis A disconfirms a rival hypothesis B to the degree to which B is inconsistent with A. So if x confirms hypothesis A, y confirms hypothesis B, and if x carries a greater evidentiary weight than y, then A is confirmed and B is symmetrically disconfirmed*» [24]. Total evidence is thus fundamentally a confirmatory approach, going for the majority rule as the answer, instead of carefully testing if some individual gene in the concatenation rejects some groupings, and identifying which ones. This is problematic as it makes the untested assumption that there is a unique phylogenetic answer, which relevantly summarizes the phylogenetic informations of the different markers. Inevitably, in a verificationist approach (for instance after the concatenation of multiple markers) the most-parsimonious tree or the most-likely one is always «well-"corroborated"» [20]. Yet, as falsificationists would point out, it does not mean that this concatenation tree has been proven to represent the true evolutionary history for all the genes. Falsificationists would claim that the «y» characters in favor of hypothesis B have been unfairly dismissed.

This is a real problem, as more sequences would increase the global bootstrap support even when conflicting in phylogeny [15]. This may be true even when disagreements between markers remain, due to lateral gene transfer, hidden paralogy or artefacts [5]. So, when provided with a long concatenation and a verificationist tree, «*systematists typically talk about 'congruent' characters, but what they really mean by that is the coherence of sets and subsets of character statements relative to an inclusive hierarchy*» [22]; the common tree whose existence was taken *a priori *for granted. Importantly, such a «*test of congruence under total evidence [...] is shown to be related to the coherence theory of truth in metaphysics and thus to coherentism in epistemology*» [22]. What this does mean is that such a method is not employed independently of an ontological conception of species, which is not necessarily explicitly stated and, should it be, would not necessarily be approved by all the community. As demonstrated by Ruse, "*as soon as one starts breaking organisms into parts, one must bring in theory ... Take two bears, one white and one brown. Do they differ in one feature, or does one take each hair separately ... The point is whether someone who explicitly eschews the theory has the right to combine all the hairs into one feature*" [22]. What the systematists who work from a verificationist/coherentist point of view classically do, is to force a unique model upon all markers, as if they were not as many gene histories as there are genes: they assume that, like bear hairs, genes can be combined in one feature.

Yet, if the hairs of a bear all quite likely share a same origin, in a context of lateral gene transfers (hidden paralogy and artefacts), the *a priori *hypothesis that there is a common phylogenetic history for the genes might be very risky to embrace, as the resulting truth might well be very artefactual. After all, even random markers concatenated together can strongly support a tree. On the one hand, "*as Farris himself pointed out: "... the decision [the choice amongst competing hypotheses of relationships] is made by accepting the stronger body of evidence over the weaker, and ad hoc hypotheses of homoplasy are required to the extent that evidence must be dismissed in order to defend the conclusion*"» [22]. On the other hand, despite what it seems "*congruence does not support a phylogenetic hypothesis, for the congruent characters need not be homologous, they could also be homoplastic*» [22]. Philosophically speaking, the least we can say is that the greatest care should accompany the positive interpretation of any global well-corroborated topology. There is indeed a high type I error to accept a wrong tree with this approach.

Some systematists, such as Kluge, came progressively to realize some of the weaknesses of verificationism, and tried to shelter phylogeny under the apparently more protective aura of falsificationism. They reworded the logic of the test of congruence and insisted on the fact that «*in phylogenetic analysis, an hypothesis of relationships can be said to be falsified if a perceived synapomorphy is inconsistent or in conflict with the hypothesis*», a claim that indeed sounds falsificationist [20]. However, there are numerous reasons for which this semantic trick falls short, as discussed in length and convincingly by Rieppel.

To clarify what was still not falsificationist in phylogeny, let us summarise very briefly how strong falsificationism functions. This popperian approach to scientific knowledge rests on what is called an "hypothetico-deductive" logic, which requires that a testable prediction is deducible from a theory/hypothesis and its auxiliaries. The famous example given by the philosopher is that of the statement «*all ravens are black", which can be logically transformed to «there is no one thing that is not black and is a raven," and that statement in turn can be logically transformed to «no white raven is here now". Hence, if one accepts the observation of a white raven here and now, then that negation of the negated observation statement contradicts the universal proposition in logical space, and falsification occurs*" [24]. Yet, there is more to falsificationism, since it is also an «*asymmetrical all-or-nothing affair*» [24]. The non-refuted, non-rejected hypotheses remain in fact unproven. Even with a reformulation, classical phylogenetic approaches fail to acknowledge this assymetry as the same fact classically symmetrically confirms a statement (if it exists), or disconfirms a statement (if it does not exist). The promotion of a concatenated tree as the true tree is still, thus, opposed to the "*asymmetry of falsification*." For a falsificationist, a tree resulting from a concatenation is never proven. Namely, "*if a prediction deduced from theory is met by experience, it does not confirm the theory, it only corroborates it. If the prediction is not met, or rather, if it is accepted that the prediction is not met, falsification of the theory occurs*" [24]. As expounded by Rieppel, '*it is evident that no such asymmetry of falsification obtains in the 'test of congruence' based on total evidence. Congruent characters (coherent character statements) confirm a hypothesis of relationships to the degree that incongruent characters disconfirm it symmetrically. This is the relationship expressed in the ensemble consistency index (or tree length)*» [22], or in a higher likelihood for the concatenation ML tree. Indeed, since most phylogenetic programs work by identifying the most likely relationships between species while eliminating the most unlikely, a best tree is simply the one having a slightly higher acceptability than the rest in the output given to the user, which usually contains a large number of very different trees. Yet, even if such a tree is still unproven, some relationships, by definition not reported on this drawing, might well have been rejected in the process of its reconstruction.

One of our guiding principle was to look for these locally rejected grouping and to try to promote a way to think more assymetrically about phylogeny without taking the verificationist shortcut, as the only answer of a phylogenetic analysis. Instead of letting our main concern be about the 'best supported' hypothesis of relationships, we decided to focus on the most falsified hypotheses of relationships. To do so, we first looked not only at the dominant relationships – the ones found on the best tree-but also at all the challenged associations and the multiple phylogenetic falsifications they allowed us to infer. Interestingly, we do not need a fully resolved tree to produce such results: the list of the bipartitions, not necessarily consistent between and within a marker suffices. Second, we analysed the refuted relationships for multiple markers of interest independently, to avoid masking any minor phylogenetic histories that would be overwhelmed by the consensus in a verificationist concatenation. We feel that this perspective might more efficiently gain taxonomic knowledge than the traditional attempt to increase the support for a given possible phylogenetic group. Indeed, from an epistemological point of view, it would allow some firm conclusions to be drawn about which groups are not true for a given dataset, while the classical approach would establish only a limited number of relationships that have not been falsified yet.

### Our approach to refute impossible groups

We wrote a program in java, named Impossible, which looks first of all for the relationships that are the most strongly falsified by the genomic data, using bootstrap bipartitions as infiles (see Material and methods for details). In brief, Impossible estimates a degree of impossibility for any pair of species or groups of species by comparing and ranking all the bipartitions, for each marker as well as for all the markers, if they are above a user-defined threshold.

An Impossible analysis can then be followed by statistical tests, which can help decide which groupings are likely statistically rejected. We suggest using principal components analysis [25] on the data reflecting the degree of impossibility for any pair of species to group together. This multivariate analysis takes the data points, which correspond to all the pairwise impossibility relationships, and transforms them onto a n-dimensional plan, where the dispersal between species is maximised. That way, in the principal component analysis, the higher the pairwise impossibility score between two species, the further away from each other they will be placed on the graph. Conversely, species for which groupings are possible will tend to cluster more closely on the graph. Observing the spread of species can thus, provide a first clue about which groupings are refuted.

Once the data have been organised (for instance along the two main axes of the principal component analysis), the two-sample T-test and the Wilcoxson rank-sum test can be applied to compare the values of the coordinates of *a priori *user-defined groups of species [26]. These statistical tests allow determination of whether two groups of data, A and B, are not significantly different and can be considered as a subset of an overall common group or not. If the data have a normal distribution, and if the means and overall standard deviations of groups A and B are not statistically different, the grouping is statistically possible. Conversely, if their means and their standard deviations differ significantly, the two groups can be determined as independent. The grouping of A and B is then statistically rejected. Clearly, the size of the data sets under investigation will affect the overall accuracy of the statistical analysis, and larger data sets of roughly equal size provide better estimates than smaller sets.

### Application to several prokaryotic genes

**Figure 1:**
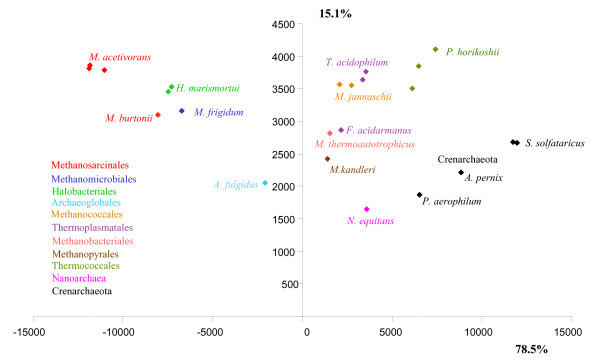
**Principal Component Analysis of the archaeal dataset**. Degrees of impossibility of the pairwise diagram were used to infer this principal component analysis. The variance explained by each axis is reported along the lines. Each point on the diagram corresponds to a species, and received the colours of the archaeal order to which it belongs, as indicated on the left of the graph.

**Figure 2:**
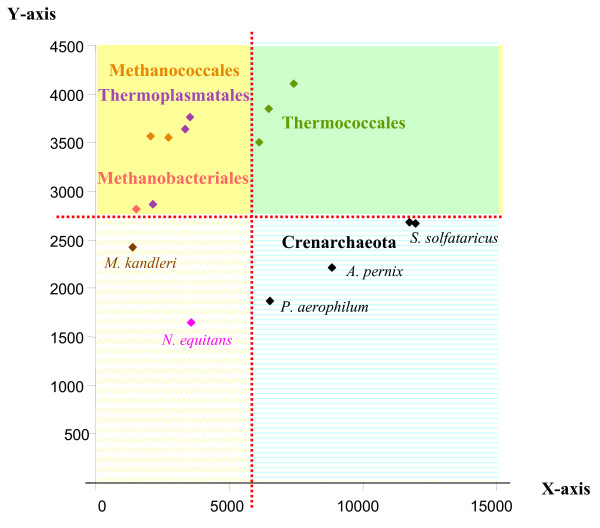
**Statistics applied to PCA results**. This figure is the same principal component analysis as figure 1, on which the boundaries deduced from the convergent results of the two-sample T-test and the Wilcoxson rank-sum test have been mapped. A thick red line corresponds to the rejection of the null hypothesis that members on opposite side could come from a same group. Identically shaded areas represent regions where the difference of coordinates would not be considered as significant.

To illustrate a possible use of our software, we considered two prokaryotic datasets. First, Impossible was used to study the mysterious phylogenetic position of *Nanoarchaeum equitans*, the sole cultured representative of the phylum Nanoarchaea, for an archaeal dataset of 23 ribosomal proteins, including 23 species, belonging to more than 10 phyla (see Material and Methods). Impossible rough estimates indicated that it was more likely that *N. equitans *should not be grouped with the Methanosarcinales/Methanomicrobiales (2723 degrees of impossibility), then not with the Halobacteriales (1461 degrees of impossibility), the crenarchaeotes (1348 degrees of impossibility), the Methanobacteriales/Methanococcales (1335 degrees of impossibility), the Thermoplasmatales (1280 degrees of impossibility) and the Thermococcales (1208 degrees of impossibility), respectively. The PCA reflects these numbers. In Figure 1, the Methanosarcinales and the Methanomicrobiales *Methanogenium frigidum *(identified as members of the Class II of methanogens in [11]), as well as the Halobacteriales, fall far to the left of *N. equitans*, suggesting that these species are not closely related. We had however no direct way to test which of the groupings of *N. equitans *with species falling close to it in the PCA, such as the Methanobacteriales/Methanococcales (identified as members of the Class I of methanogens in [11]), the Thermoplasmatales, the Thermococcales or the crenarchaeotes, were rejected or possible. We thus used these 4 different *a priori *groups, each containing more than three species, to operate a "triangulation" and define which could not be closely related to the Nanoarchaea. Based on the coordinates of each species on each axis of the PCA independently, we used the two-sample T-test and the Wilcoxson rank-sum test at the level of 5% to test if some associations amongst all the possible pairs of these 4 groups of species were significantly rejected. Interestingly, the two tests convergently concluded that the coordinates of the crenarchaeotes and the Thermococcales along the first axis were significantly larger than the coordinates of both the Thermoplasmatales and the Methanococcales/Methanobacteriales. We symbolized this result on Figure 2, by a vertical frontier between these two groups of species, as the tests indicated that the species on each side of the frontier (i.e. the crenarchaeotes and the Thermococcales on one hand, and the Thermoplasmatales and the Methanococcales/Methanobacteriales on the other hand) were statistically different. When we repeated the same tests for the y axis, we observed a second frontier, horizontal, separating the "lower" crenarchaeotes from the "higher" Thermococcales/Methanobacteriales/Methanococcales/Thermoplasmatales. The conjunction of these two frontiers remarkably isolated *N. equitans *from the 4 groups, but left it in the vicinity of *M. kandleri*. This analysis thus indicates that, contrary to previous suggestions, *N. equitans *might not represent a third archaeal kingdom [27,28], nor a Thermoccocales [29], but clusters within the euryarchaeotes, possibly as a relative of the Methanopyrales. This proposition now deserves careful consideration to ensure that it does not result from a long branch artefact, since both *Nanoarchaeum *and *Methanopyrus *display high evolutionary rates for the components of their transcription apparatuses [30]. The proximity between Methanobacteriales/Methanococcales and Thermosplasmatales in the upper left corner of the diagram, delineates another possible group. It is also consistant with phylogenetic analysis of concatenated ribosomal proteins that weakly groups Methanococcales and Methanobacteriales together, and the lack of any support in the backbone branches separating them from Thermoplasmatales, which suggest that a specific sister-grouping between them is indeed possible. However, so far no specific synapomorphies appear obvious. Other groupings, such as the two distinct classes of methanogens identified in a previous study [11], are similarly not rejected by Impossible. Methanococcales, Methanobacteriales and Methanopyrales fall on the righthand side of the x axis in the PCA (class I methanogens) as Methanosarcinales and Methanomicrobiales fall on the lefthand side (class II methanogens) (Figure 1).

**Figure 3:**
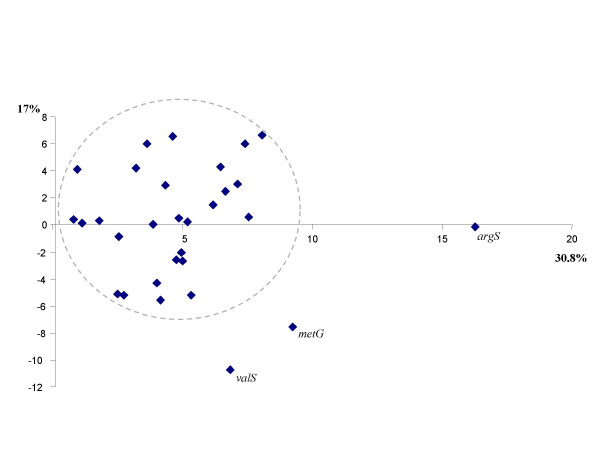
**Principal component analysis of the 31 prokaryoticcore genes**. This PCA of core genes is based on their degrees of impossibility for the 58 most commonly rejected bipartitions, as reported in the .pharm. In grey, we have drawn the cloud of markers without obviously different phylogenetic signal. The variance explained by each axis is reported along the graph.

**Figure 4:**
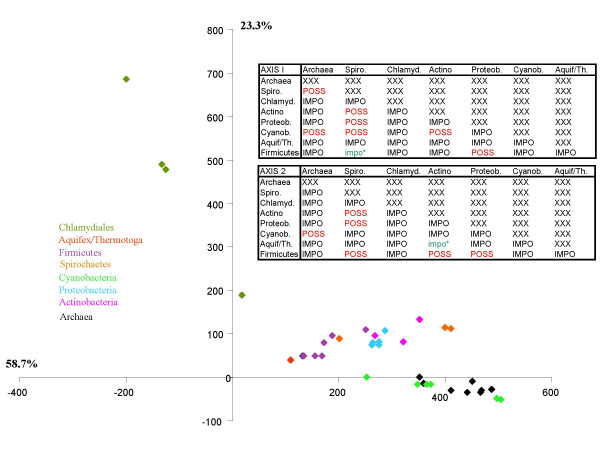
**Principal component analysis of the degrees of impossibility of 43 species, for 28 core genes**. Each point on the diagram corresponds to a species, and received the colours of its group, as indicated on the left of the graph. The two tables contain the results of the two statistical tests applied to the coordinates on axis 1 and on axis 2, to decide if *a priori *groups of points on the principal component analysis are overlapping or not. Eight groups were tested (see. Mat. and Meth.). When two groups were non-overlapping, we reported IMPO for impossible grouping on the table, and when we could not reject the null hypothesis that they overlap, we reported POSS. We indicated by an impo*, when one test only rejected a grouping.

Another prokaryotic dataset of 31 ubiquitous markers constituted our second case study, as the classification of prokaryotes remains a major challenge for phylogeny [31]. These genes, present in no less than 180 complete prokaryotic genomes [12], are a potentially important set of molecular candidates to analyse what might be the vertical backbone at the "Tree of Life" level. In order to reduce the potential impact of lateral gene transfer or other artefacts that would contribute an important non-vertical signal, we carried out a first Impossible analysis focusing on the congruence between these 31 markers. We performed a principal component analysis, based on the degrees of impossibility for the 58 most commonly rejected bipartitions presented by the Impossibility diagram. Figure 3 shows that all these markers belong to a cloud with the possible exception of *argS*, *valS *and *metG*. We subsequently removed these 3 genes from our analysis and reran Impossible with the same parameters on the 28 core genes for which no incongruence was suspected *a priori*. The second principal component analysis based on the degrees of impossibility of the 28 markers and for 43 species is reported in Figure 4. In this diagram, the species known *a priori *to belong to a monophyletic group tend to group closer (i.e the Proteobacteria are very close on the diagram), but the distance between the different monophyletic groups varies (i.e. the Proteobacteria are closer to the Actinobacteria than to the Chlamydiales). Some phylogenetic information might thus be gained from this principal component analysis. The tables in Figure 4 report when this dispersion in the principal component analysis was considered statistically significant and excluded that two groups belong to a common clade. Interestingly, the two axes provided highly congruent answers concerning which groups were rejected or possible (23 agreements and only 5 disagreements). Unfortunately, the position of the Chlamydiales remains undecided. Their isolation from the other bacterial taxa under study is made obvious on the PCA (Figure 4). Consequently, the Chlamydiales are involved in a very high number of rejections of potential relationships in the group-group diagram (data not shown but see tables in Figure 4). This could be because all known representatives of this group are obligate intracellular pathogens, a lifestyle that can result in drastic changes in evolutionary rates and codon uasge biases. Additional slowly evolving representatives of the Chlamydiales appear to be needed to understand the origin of this group and no concatenated trees involving these taxa, wherever they branch, should be taken for granted. It is interesting to note however, that despite their fast evolutionary rates, they do not artefactually group with distantly related species (i.e. Archaea), as would be the case due to typical LBA artefact in a molecular phylogeny.

The non-conflictual information about the rejected groups and the convergent information about the possible ones allowed us to suggest three possible groupings: (i) the proximity of the Archaea and the Cyanobacteria, (ii) the proximity of the Spirochaetes and the Actinobacteria and (iii) the proximity of the Proteobacteria and the Firmicutes, while other configurations are rejected. The possibility of an even larger clade comprising this last two could also be put forward, consistent with the dispersion along the principal component analysis. These results are of interest as they suggest an early emergence of both Cyanobacteria and Archaea, should the root of this tree be placed in between the two prokaryotic domains. Should the tree-like reasoning apply to these markers, this hypothesis would be consistent with the proposition that there might have been methanogenic Archaea and Cyanobacteria living contemporarily 2.8 billions years ago [32,33]. Such an ancestry of Archaea is however importantly contested by T. Cavalier-Smith [34], for whom the old fossil evidence is too indirect to be conclusive. Presently, Cyanobacteria live closely associated with halophilic Archaea in microbial mats found in saline environments, and it has been suggested that the fact that both Cyanobacteria and Archaea display gas vesicle structures (which help them keep afloat in their natural environments) could be the result of lateral gene transfer. Yet, on the other hand, if the relationship between these taxa is one of common descent, this feature could be instead an ancestral characteristics of this group. Further in-depth phylogenetic studies will be needed to decide if this connection is genuine or should be rejected. *A priori*, this grouping is unlikely to result from an artefact due to preferentially shared lateral gene transfer, as prokaryotic groups known to have undergone frequent interdomain gene transfers (such as Thermotogales or Methanosarcinales) are not artificially attracted to representatives of the donor domain in our PCA (Figure 4). Furthermore, earlier gene phylogenies dealing with partially overlapping sets of markers never identified such a problem [35]. Yet, it might reflect another problem if one considers the proximity of Thermotogales and Aquificales (Figure 4), which are both hyperthermophilic specialist bacteria with small genomes, as artefactual. Perhaps similar lifestyles could impose similar evolutionary biases on their genomes [35]. Finally, the possible proximity between the Spirochaetes and the Actinobacteria, as well as between the Proteobacteria and the Firmicutes also deserve to be studied in evolutionary scenarios, as it might help to refine our understanding of the bacterial evolution.

## Conclusion

Surprisingly, the application of our simple software based on a slightly modified perspective allowed us to extract some original information about complex phylogenetic problems. We easily rejected a large number of *a *priori possible combinations, which is our primary results. That some groups are left possible for these markers is then mentionned, as a complementary result. None of these possible group (the proximity of N. equitans and of the Methanopyrales, of the Archaea and the Cyanobacteria, the possible groupings of Methanobacteriales/Methanoccocales and Thermosplasmatales, of Spirochaetes and the Actinobacteria and of the Proteobacteria and the firmicutes) should be taken as proven by this approach. Instead all of them should be tested further under the advice of biologists working with the organisms, with other markers or characters, and eventually be falsified. In any case, we hope that our present work encourages the attempts to multiply the perspectives in phylogeny. In this respect, a lot remains to be done to investigate more exhaustively, and with more powerful tools than ours, the "impossible informations" carried by phylogenetic markers, and their conflicting signal, which is too rarely discussed (but see [36] and [37] for interesting exceptions).

## Competing interests

The author(s) declare that they have no competing interests.

## Authors' contributions

JB wrote Impossible. YB provided the biological expertise required to interpret the phylogenetic results and helped with the redaction of the manuscript. EB conceived the project, supervised it, created the datasets and wrote the manuscript.

## Reviewers' comments

*We would like to thank the referees for their comments that greatly help to improve this manucript*.

### Reviewer's report 1

Mark Ragan, The University of Queensland, Brisbane, Australia

This is an interesting paper that challenges us to consider current practice in molecular phylogenetics (specifically, the testing of phylogenetic relationships) in the context of competing theories of epistemic philosophy. The authors ignore Bayesian approaches, in which the empirical relative frequency of a topology (determined by sampling from the equilibrium posterior probability distribution) converges to its corresponding marginal posterior probability given the prior probability distribution, model and data. Instead, relationships are viewed completely through the lens of the likelihood approach and its limiting case, parsimony (hence the authors' concern with synapomorphies at Background, paragraph 2). These approaches do not yield estimates of the probability of bipartitions; rather, the robustness of a clade is typically assessed by use of the bootstrap.

Philosophical theorising on classification is as old as Plato, and will continue for centuries. In the absence of logical error, the authors' programme cannot be proven either right or wrong, although it might be examined (subjectively) for aesthetic qualities such as elegance. Thus I offer these comments to help explore not the truth of falsity of their claims, but rather their generality, explanatory power, and utility in application to real problems.

(1) Does concatenation practice rest on verificationism? The authors assert that when bootstrap values are low, phylogeneticists "try to increase the support of the clades under study by providing always more phylogenetic signal (*i.e*. increasing the sequence length)". No doubt some do, but alternatively we might view sequence regions as samples of the genome. A small sample (*i.e*. a single short sequence) might represent a region of the genome that happens to be anomalous in some way – as the result of mutational saturation, selection, GC bias or whatever – and it would simply be good scientific practice to obtain further samples from different genomic regions, in hopes of integrating over as much local misinformation as possible. The motivation would be not purely to pull signal out from stochastic noise, but equally to avoid interpreting a local anomaly as the true genome-wide phylogenetic signal. This seems beneficial regardless of philosophical programme. Indeed it's not always clear that the verificationist and falsificationist programmes can be cleanly separated. Consider the following (admittedly not very general) case:

To test the hypothesis "there are exactly 10 thousand lilies in this 1-ha field", would I rather have data from a single random 1-m^2 ^sample grid, or from 1000? Unless the field were fully tiled with grids and I can count the lilies in every one, I cannot guarantee that I can definitively test the hypothesis. But under either programme I'm better-off with 1000 grids than with one: if the count exceeds 10 thousand (which, given the size of lilies, is more likely to happen in 1000 grids than in one) then the hypothesis is strongly falsified; and if not, I can use the number of lilies the 1000 grids do contain to assign a more-accurate probability to the truth of the hypothesis. Either way, it's in my interest to have access to more grids.

***Author response***: *I want to thank the reviewer for his very interesting comments. It seems to me that the sample perspective is interesting. I will embrace this analogy in my answer, and I would argue (i) that falsificationism and verificationism approach can still be distinguished, and (ii) that the a priori hypothesis that there is a unique field to sample is coherentist and is not a free assumption, as it biases the meaning of the results of the multiple sample practices*.

*It is indeed very important to have a better estimate of what the field of 1ha contains (of what the tree of life is), if there is such thing as a 1ha field (such a thing as a tree of life). If there is no such thing, but several overlapping patches of grass, multiple fields in this 1 ha area, then our estimate based on multiple sample will certainly be accurate, but it will not be a relevant description of the distribution of lilies. In fact, the number of lilies in each patch could differ for the simple and good reason that there is no such thing as a unique homogenous 1ha field, and then why would be willing to go on and comment about how rich this field is? So if the background hypothesis is correct, we are better off with more estimates, but we should make sure that we test that the hypothesis is safe first. We can not only be verficationist*.

*However, in phylogenetic concatenations, if someone assumes that there is a tree, he is not prone to consider that different estimates could genuinely be different and that these differences should be tested to validate the concatenation approach itself*.

*Where the falsificationist and verificationist approaches could differ then is that while the verificationist would assign itself the goal to describe **the **field as well as he can, the falsificationist would eventually be happy enough to reject 1. that there is a field, 2. that there is this number of lilies in this patch. The first one is coherentist (one field to count them all), the second one does not have to be so (as many differences as we can report could undermine the claim that there is one field to be discovered out there)*.

(2) In relationship-testing, would a falsificationist be "more likely [to] be interested by the rejection of impossible groupings, underlining what can not be a natural group"? For Popper, the possibility of falsification demarcated science from pseudo-science (see *Conjectures and Refutations*). One exposes a hypothesis (here, a proposed clade) to a risk (a test in which the proposed clade can be shown to be false); if the hypothesis survives, its scientific status is enhanced (if it fails, it might be rescued by *ad hoc *measures, but would come out with diminished scientific status). Falsification in this sense seems quite different from what the authors propose, which seems more a matter of chipping away at the space of possible hypotheses. Do the authors imply that by refuting some dozens (or hundreds, or thousands) of clades, all remaining un-refuted ones (or the refutable ones among them) are increased in status?

***Author response***: *No, we do not mean that. We stick to the deepest spirit of refutation, which is not about proving anything, but disproving some things*.

For large datasets, astronomical numbers of possible clades will remain, most of them untouched by human imagination, but each now with an infinitesimally increased status. Or (as in the *Nanoarchaeum equitans *example) must we happen to care about the individual clades that happen to be refuted?

***Author response***: *No increase in status for us. What was not rejected remains unproven for us. We simply suggest that we should keep in mind what was rejected and make it visible. Then, if someones wishes to test the coherentist hypothesis that Nanoarchaeon is a X, we could at least tell him what are the many evidence that actually opposed to this claim*.

(3) What does "falsificationist-lite" mean? In their "Impossible" analysis, have the authors not fallen into what Rieppel called the "semantic trick" of superficial falsificationism, where clades with little or no observed bootstrap support are considered refuted? Is this really falsification, in either a strong or weak sense? Do the authors recommend rejecting one pre-identified clade each time, or are they carrying out multiple tests?

***Author response***: *Falsificationist-lite means falsificationist in spirit, more eager to disprove than to confirm, to stress the irreducibility of conflict than to hide it. We advocate this approach not because falsificationism sensu stricto would be a better approach than verificationism for phylogenetics (both could not actually be applied in their strong sense), but because this perspective can complement the verificationism of phylogenetic methods too prone to look at what the majority signal is, and not to look at what the plurality signal is, and would reject. Here, in this modest approach, we intend to provide a tool for anyone who, once done with his individual phylogenies, wants to look at the data in a way that he could conclude easily enough about the falseness of some (could be multiple) pre-identified hypotheses of relationships. So, in our approach there is everything that is in the classical approach (a simple use of bootstrap support to reject some groupings, but a refusal to use it to affirm that a grouping is proved) and a little bit more (a complex use of multiple bipartitions together to deduce the degrees of impossibility of a given relationship). It means that this approach is not falsificationist in the strong sense and that there is room for improvement in this respect to make it even more complementary to traditional approaches. It would be valuable for instance to evaluate what was never ever seen in any trees, for any gene, not even ever suggested to be a grouping, without resting on bipartitions, but maybe directly looking at the pattern not inferred for each site in an alignment*.

(4) Is lateral genetic transfer really the "most pervasive problem" in large-scale phylogenetics? There is little evidence that LGT is a problem at all for many taxa that researchers care about (*e.g*. animals, green plants, fungi). A stronger case might be made for the problematic pervasiveness of model mis-specification, methodological inconsistency, lineage-specific biases, long-branch attraction, paralogy, or (for systematics) the grade-*vs*-clade issue.

***Author response***: *Certainly, those are problem as well. Hence my claim deserves to be tested, but can not be tested with tools that suppose that there is a unique tree a priori, and hidding the conflicting signal instead of highlighting it. One reason for which few transfers are observed by phylogeneticists using classical phylogenetic approaches is simply that they can not detect the vast majority of these lateral events. The recent works of Brochier et al. is an example of that: these authors (hello Celine and coworkers) simply check if concatenated trees agree with each other and from that compatibility conclude that there is no LGT in their whole dataset. However, by doing so they simply acknowledge that they manage to obtain a dominant signal, which has not to be identical in any of the genes they used for concatenation. All these genes could support phylogenies that differ locally in topology, for instance because of LGT. The reason why the reviewer mention some presumably safest groups (plants, fungi, etc.) could then be partly psychological. Because some people believe that there are mechanisms causing LGT that could be important in prokaryotes, they sometimes proceed to some further phylogenetic analyses, which generally suggest that phylogenetic markers are too weak to tell about the extent of LGT, or even show some conflicting signal. Yet, for eukaryotes, few people have such an a priori belief, so few test it. Hence, they can not see LGT, and not see it as a problem, and they would keep thinking that artefacts are a potentially larger issue in their dataset. But, should some of them start thinking that protists acquire genes laterally, as suggested by Andersson, then we might start seeing more reports of LGT in those safest groups too. I feel this is a limit of many of Philippe's et al. publications including protists in the analyses (hello Herve and coworkers). Because they use long concatenation, they get nicely resolved trees but those trees do not tell us anything direct about the true phylogenetic history of individual markers, and if some LGT were dismissed to got the emerging picture. So honestly, I would say it is an open question at large evolutionary scales (when protists and prokaryotes are involved), and to make our mind about it more correctly, we will need to keep our eyes open to the discoveries of microbiology and molecular mechanisms of LGT. Also, I meant more pervasive because LGT undermines the very existence of a tree of life, while artefact just make it harder to be discovered*.

(5) Is the posing of Cavalier-Smith's cabozoan hypothesis really an illustration of testing multiple possible clades? Cavalier-Smith is a powerful exponent of the position that both clades and grades are important in systematics. This position was once almost axiomatic, then fell into eclipse with the rise of the strong cladistic programme. One suspects that Cavalier-Smith had more in mind than alternative topologies when posing the cabozoan hypothesis; the "contrast of phylogenies" may be a key test among alternatives, but for him, there can be other key tests that are not topological in nature.

***Author response***: *You are certainly right. Tom Cavalier Smith proposes indeed grades and clades (and I personally approve of this way of looking at things for classification purposes). Also I would assume that in many cases he actually means clade by clade (even if his own favorite topology (classification framework) can change), hence these claims (and singularly this one) could sometimes be tested topologically (among other approaches)*.

(6) In what sense is comparison with other gene phylogenies different from total evidence, i.e. verificationism? In the third paragraph under Results & Discussion, the authors comment that weakness of phylogenetic markers (*i.e*. their lack of resolving power, illustrated here by rRNA sequence data) can hinder tests (including attempted falsification) of theories. If even robust results may be erroneous, what is to be done? The authors recommend comparison with other gene phylogenies. This may not be total evidence, but it does involve recourse to additional information; in what way does this move beyond verificationism?

***Author response***: *The approach is different and so could be the results. Genes are analysed individually as well as collectively: so individual dissent can be identified, local congruences can be detected, so that the phylogenetic signal does not have to be coherent at the end of the analysis. It may well be the case that no common possible "groups within groups" struture emerges at the end of such an analysis, and that we end up observing multiples patterns of rejection: coherent between some genes only and incoherent with any other gene, while this is impossible to report in a verificationist approach. So this differs from total evidence in the sense that the goal is not to maximise the degree of confirmation of a single syncretic hypothesis*.

(7) Do concatenation trees represent the true genomic history? The principle embodied in the quotation leading off the third paragraph of the section on Epistemological principles ("inductive support works symmetrically...") should, I believe, be credited to Kolmogorov. That aside, one does not have to be a falsificationist to ask when, and indeed if, datasets should be concatenated (the total evidence approach *in sensu *Kluge); this debate permeates the supertree literature. More fundamentally, Ford Doolittle has asked whether genomes necessarily have phylogenies (his analogy with city telephone directories). Reducing all of this to a battle between Carnap and Popper does injustice to the underlying complexities, both biological and algorithmic.

***Author response***: *Indeed, yet I feel it gives a solid rationale to preach in favor of the diversification of methodologies and scopes in phylogenetics, a fields that tend to be too much concerned by the question "what is the tree of life?" and not by the other most fundamental essential questions: "is there a tree of life?" and "what if there is no such Tree?"*

(8) Are species relevant? In the section on Epistemological principles, paragraph 4, why do the authors relate tests of 'congruence' to an ontological conception of *species*, as opposed to an ontological conception of *genome*? We are talking about nucleotide (or protein) characters, not bear hairs. In what relevant ways might the two conceptions differ?

***Author response***: *It is related I believe in many people's mind as the use of a single phylogeny to find the single true phylogenetic position of a species is a coherentist approach. This means that some assume that they will somehow end up equating the genome histories with a single place and time along the tree of life structure (the species origin). This conception of a species is indeed not relevant to me, as I am more inclined to conceive species as emergent cohesive arrangements of micro-components with their own histories, which can themselves trace back to different "ancestral" systems at different times and different places in the past. I am thus more interested in trying to test how many genes can be proved to coevolve (to have a cohesive pattern of inheritance). If it happens that we have no evidence against the cohesion of all the markers under study (i.e. no different impossible groupings derived based on these markers), then we could start wondering what their common tree is. Otherwise, we should prove that there is a common phylogeny for species to be inferred. In this respect, Impossible analyses is a good first step to test without a priori if a series of markers should be combined and which ones*.

(9) What body of observation or theory might inform the user's choice among linear, multiplicative and exponential weighting (particularly given the non-independence of nodes within a given tree)? Of bipartition support threshold?

***Author response***: *Because the issue of significativity is not specific to our software (who can tell what is the bootstrap value above which a group is **significantly **supported?), I can only think about being empirical. Here, we offer the user different optional settings so that he tests their possible influence, to use a metaphor to try to zoom in and zoom out the distances between taxa and figure if the dispersal on the PCA for instance is dramatically modified with some parameters choices, or remain stable. Then, the user should report that such and such groups where impossible given such and such tresholds*.

### Reviewer's report 2

Eugene V Koonin, National Center for Biotechnology Information, NIH, Bethesda, Maryland, USA

This paper offers a fresh and interesting view of the epistemology of phylogenetic analysis. The authors start by explaining that traditional phylogenetic analysis is verificationist in its epistemological foundations (inevitably, this brings to mind Moliere's Monsieur Jourdain who discovered, to his great delight, that all his life he spoke prose... but nevertheless, this is an important clarification on the nature of the methodology that is applied non-critically all too often) and develop the alternative, falsificationist (Popperian) paradigm. They then describe crude (by self-admission) software they developed to falsify (prove impossible) phylogenetic affinities and present some examples of phylogenetic inferences made within this new framework.

The gist of the paper is, obviously, philosophical, and I will make some comments in the same spirit, i.e., without getting into details of the actual method and examples.

It seems to me that the authors under-appreciate (or at least do not present explicitly enough) the complementarity/conceptual equivalence of the verificationist and falsificationist approaches.

***Author response***: *We have modified the text to make this complementarity explicit*.

Indeed, when a phylogeneticist embraces the falsificationist credo, she can (at least, in principle) refute all possible tree topologies, except for one, and thus, effectively, ***verify ***the only remaining topology. This is becoming obvious in the case of 4-species tree: to verify one topology, all it takes is to falsify the remaining two topologies. Of course, in practice testing all topologies very quickly becomes prohibitively expensive with the growth of the number of nodes. However, refuting all positions of a given branch except one is still possible. In this respect, I think it is desirable to discuss some existing phylogenetic tests, such as Adachi-Hasegawa and Shimodaira-Hasegawa: what is their epistemological status?

***Author response***: *Even if in principle it is absolutely possible that refuting 2 out of 3 hypotheses could allow to establish that the one that's left is the true one, I must stress on the fact that there is no need that such a coherent pattern is to be observed. It is also possible that all the three topologies could be rejected by different markers (in this case there is no common best tree). In absence of a common best tree, the issue becomes then to identify which sets of markers were cotransmitted, i.e. because they share common patterns of rejection and thus allow common possible groupings to be defined*.

*The status of the tests based on the comparisons of multiple test topologies (i.e. Adachi-Hasegawa and Shimodaira-Hasegawa) is interesting. We published elsewhere (Susko et al., 2006) that these tests have a high type I error (a tendency not to reject a wrong tree). But, to put it in a more "philosophical" phrasing, these tests evoke the Sherlock Holmes logic of inquiry: ""Eliminate the impossible, and whatever remains, however improbable, must be the truth." The potential problem here is thus to make sure that a proper set of hypotheses is being tested: if we fall short when considering our test topologies for these tests, we will end up picking the best of a bad lot*.

The author seems be getting into a bit of a philosophical muddle by tacitly equating verificationism and the principle of complete evidence. To the best of my understanding, these are connected but not inseparable. The more practical side of the same issue is the emphasis that is being made in the manuscript on the sequence concatenation methods in connection with verificationism. Perhaps, concatenation is, indeed, relevant when it comes to complete evidence. Verificationism, however, seems to be a much more general principle that permeates all modern phylogenetics (although see above) and, as far as I understand, fully applies to single-gene phylogenies. I think it would be useful to be a little clearer on this point.

***Author response***: *The referee is right. The reconstruction of single-gene phylogenies is verificationist*.

The above points are philosophical quibbles whose aim, in part, is to show that the reviewer understands the epistemological issues addressed in this paper (I do not know how well it works). Here, however, I come to the only problem that, to me, to some extent undercuts the value of this otherwise very interesting and promising paper. While the entire work is about falsificationism and its merits, the authors make a very strange, almost paradoxical somersault when it comes to conclusions and present, mostly, claims on putative new phylogenetic affinities that look suspiciously verificationist. It does not help that, when one looks into specifics, each of these connections looks dubious (granted, the authors are careful in pointing out that these are very preliminary indications). In my opinion, it would be much more appropriate to make conclusions on the falsification of some affinities that have been previously considered valid on the basis of verificationist phylogenetics or other evidence. Only then, as an addendum, some potentially interesting affinities that could not be falsified might be mentioned.

***Author response***: *The referee is entirely right. Our primary results were the refutations of groups: that some groups are left possible for these markers is mentionned, as a complementary result. Yet, none of these possible group should be taken as proven by these approach, instead all of them should be tested further, with other markers or characters, and eventually be falsified. How dubious these connections are, based on those genes, is however really difficult to evaluate, and that it is why we report them for further test. If we compare the impossible groups with some former propositions (for instance by pruning the tree of life recently published by Cicarelli et al. from the groups we were missing), we observed that the group of Spirochaetes and actinobacteria (supported by less than 40 % of BV) is not rejected, while the group of cyanobacteria/aquifex-thermotoga (supported by 40 to 80 % of BV) is rejected*.

### Reviewer's report 3

J Peter Gogarten, University of Connecticut, Storrs, CT, USA

This reviewer suggested we quote the important following publications: Hendy, M., and M. Penny. 1993. Spectral analysis of phylogenetic data., J. Classif. 10:5–24.; Lento, G. M., R. E. Hickson, G. K. Chambers, and D. Penny. 1995. Use of spectral analysis to test hypotheses on the origin of pinnipeds. Mol Biol Evol 12:28–52.; Zhaxybayeva, O., J. P. Gogarten, R. L. Charlebois, W. F. Doolittle, and R. T. Papke. 2006. Phylogenetic analyses of cyanobacterial genomes: Quantification of horizontal gene transfer events. Genome Res.; and Zhaxybayeva, O., P. Lapierre, and J. P. Gogarten. 2004. Genome mosaicism and organismal lineages. Trends Genet 20:254–260. He also suggested we comment on the possibility that nanoarchaea would be sister group to all the euryarchaea. Our results reject this possibility and suggest that nanoarchaea are within the euryarchaea.

## Supplementary Material

Additional File 1 A pdf document with a representation of what basic and combined impossibilities mean, some outfiles generated by the program (the impossibility diagram, the group-group diagram, the species group diagram, and the pairwise impossibility diagram). Click here for file

Additional File 2 A text presenting an example of the calculation of the weight of a combined-impossibility. Click here for file
