# Supplementary material for: Refuting phylogenetic relationships
Source: Biol Direct. 2006 Sep 6;1:26. doi: 10.1186/1745-6150-1-26 (PMC1574289; doi:10.1186/1745-6150-1-26)
Supplement: Additional File 1 — A pdf document with a representation of what basic and combined impossibilities mean, some outfiles generated by the program (the impossibility diagram, the group-group diagram, the species group diagram, and the pairwise impossibility diagram). [file 1745-6150-1-26-S1.pdf]

A feeling of the notions of “basic” and “combined” impossibility

A “basic” impossibility:
(1,2) are not with (3,4,5,6)
which we deduce from:
** . . . . > user defined threshold

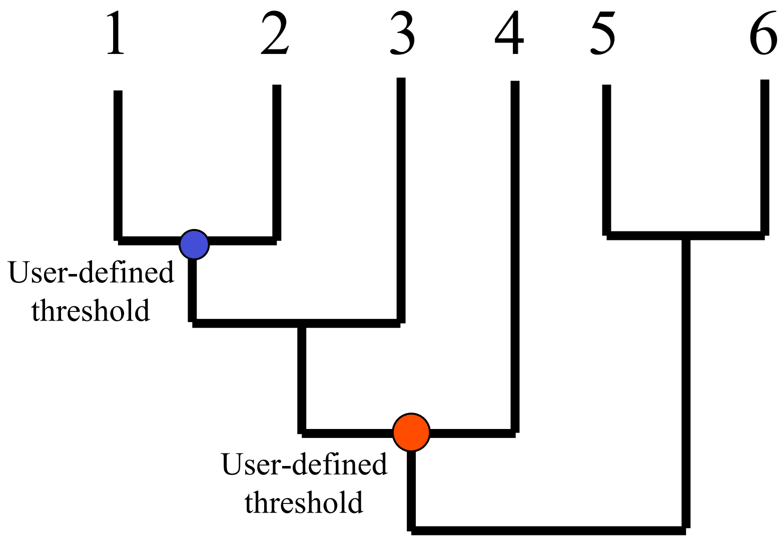

A “combined” impossibility: +
(1,2) are not with (5,6)
which we deduce from the comparison of multiple bipartitions:
** . . . . > user defined threshold
and
**** . . > user defined threshold

An example of an impossibility diagram

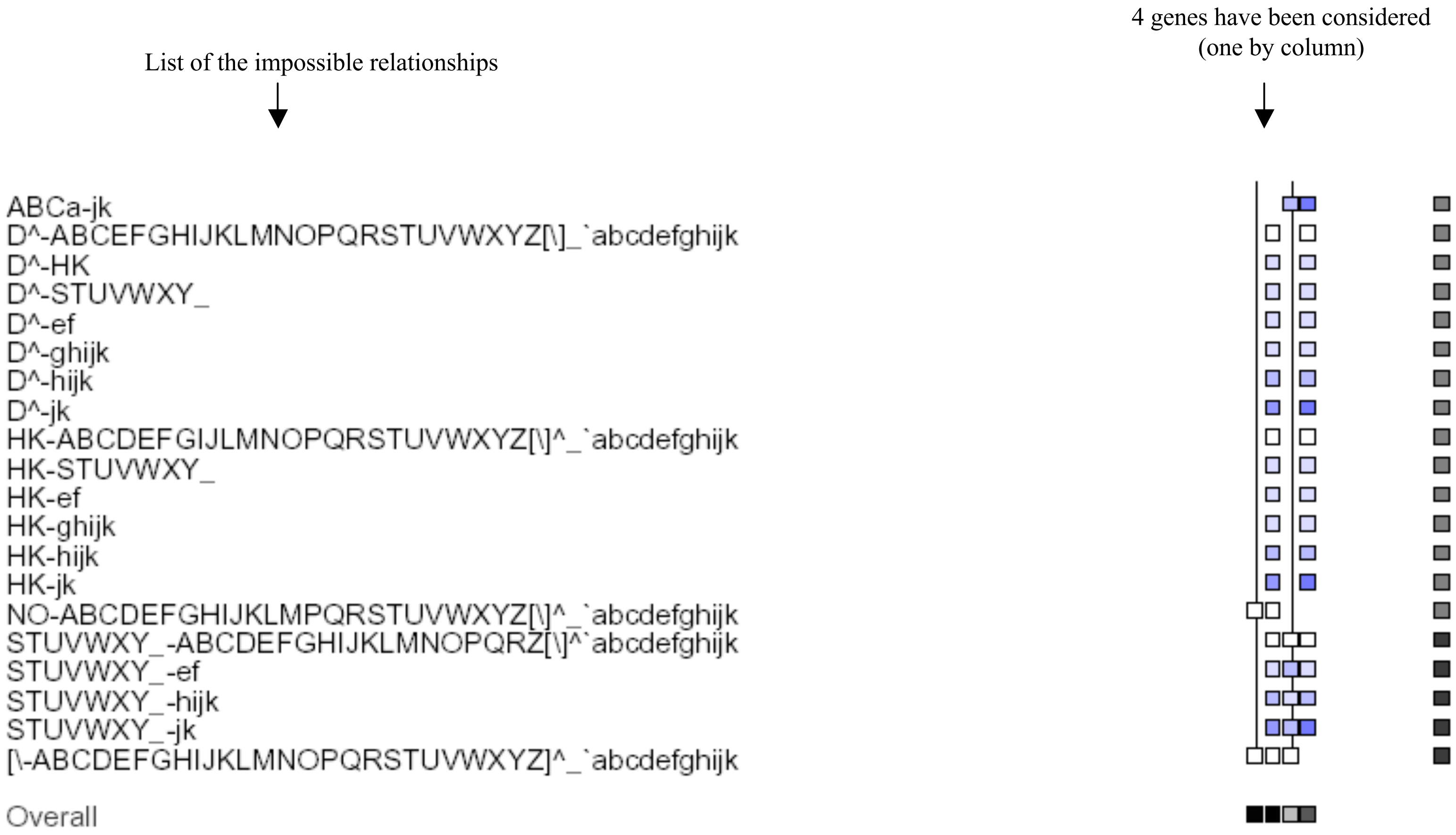

The '-' split two sets of organisms that should not be grouped together
The darker the square the most unlikely the relationship is
No square: the relation is possible according to that gene

An example of a group-group diagram

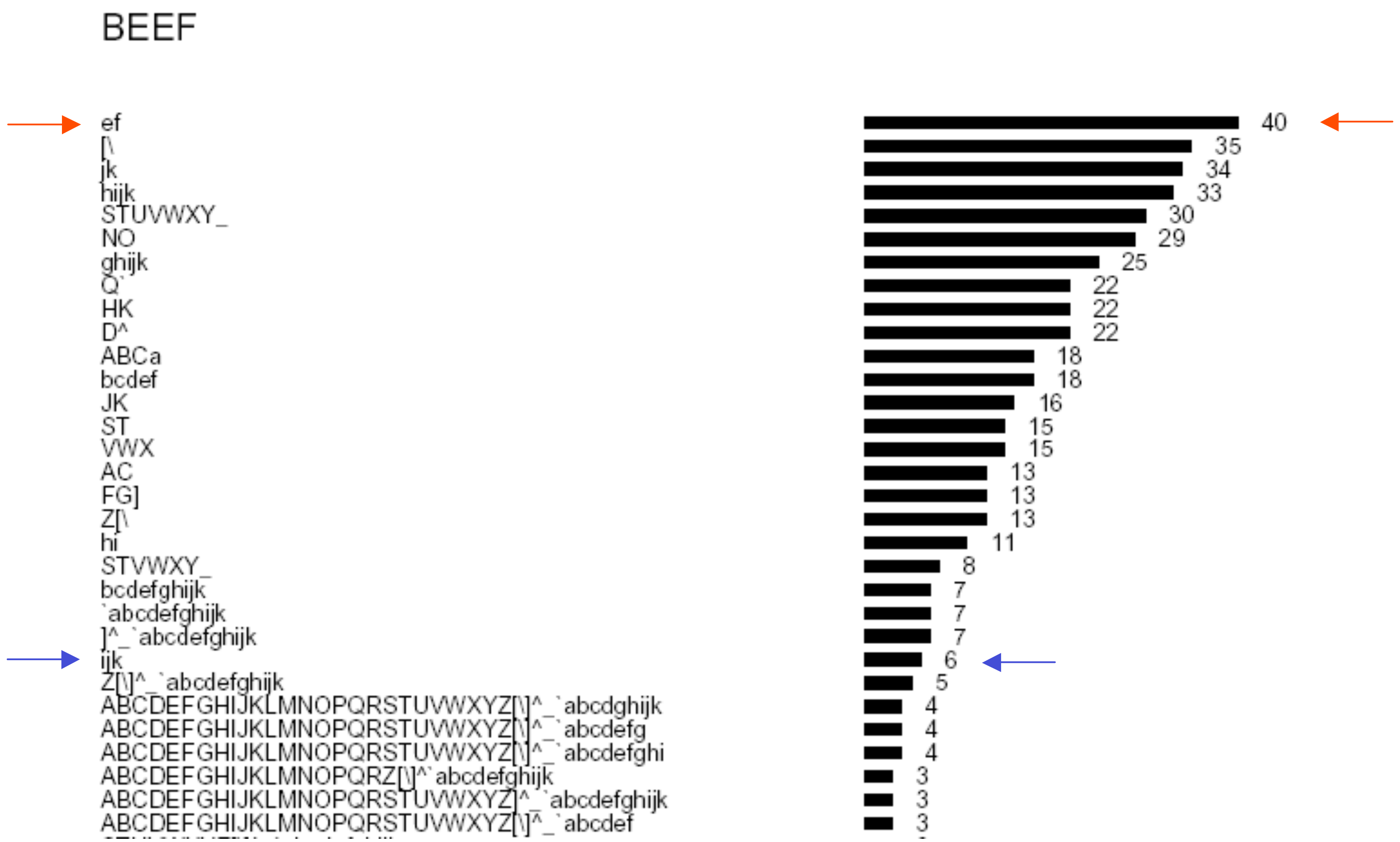

The relationship between ef is the most rejected. Its arbitrary degree of impossibility is of 40 ijk by contrast is only weakly rejected (6) over all the bipartitions with a support larger than a user defined threshold.

An example of a species-group diagram

Z PIG

[\	3
VWX	3
ST	3
bcdef	3
STUVWXY_	3
D^	2
bcdefghijk	2
Q`	2
]^_`abcdefghijk	2
`abcdefghijk	2
HK	2
ABCa	2
hi	1
ABCDEFGHIJKLMNOPQRSTUVWXY	1
INO	1
ABCDEFGHIJKLMNOPQR	1
AC	1
STVWXY_	1
ABCDEFGHIJKLMNOPQRSTUVWXY]^_`abcdefghijk	1
ijk	1
FG]	1
ABC	1

The impossibility grouping of the species “Z” within any of the listed group receives a certain value.
The higher the number the most rejected the relationship.
That way it is easy to evaluate for a species of interest with which other it would not group.
(PIG stand for pairwise impossible grouping)

An example of a pairwise impossibility diagram

OAT

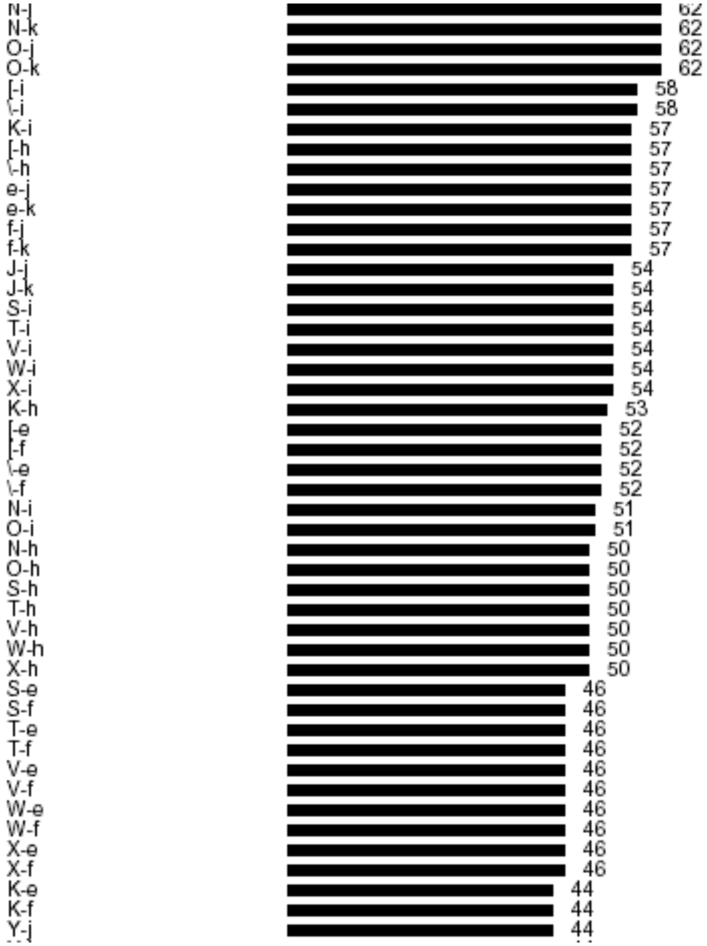

The larger the value the least closely related the species are
