# Supplementary material for: Refuting phylogenetic relationships
Source: Biol Direct. 2006 Sep 6;1:26. doi: 10.1186/1745-6150-1-26 (PMC1574289; doi:10.1186/1745-6150-1-26)
Supplement: Additional File 2 — A text presenting an example of the calculation of the weight of a combined-impossibility. [file 1745-6150-1-26-S2.doc]

For instance, if we had the following strongly supported bipartitions : ABCD|EF, ABC|DEF and AB|CDEF, since there are six different pairs, there are a total of 12 (6*4/2 ) candidate pairs for combined-impossibilities (ABCD-ABC, ABCD-DEF, ABC-AB, ABCD-AB, ABCD-CDEF, ABC-CDEF, EF-ABC, EF-DEF, DEF-AB, EF-AB, EF-CDEF, DEF-CDEF) among which only four are valid (EF-ABC, EF-AB, AB-DEF, AB-EF). To determine say, AB-EF’s degree, we would identify all of the related sets for these two members. For the AB subset, there are two: ABC and ABCD. Likewise, for the EF subset, there are another two: DEF and CDEF. In this case however, there would be several overlaps between the related sets: ABC overlaps CDEF, ABCD overlaps DEF, and ABCD overlaps CDEF. The ordering of related sets of AB and EF, as well as the estimates of the number of overlaps that they contribute to could be summarized as (ABCD [2], DEF [1]) and (CDEF [2], DEF [1]), respectively. We would then remove ABCD and CDEF, as they were both the largest groups and the groups with the most overlaps. Removing ABCD and CDEF, would leave us with two non-overlapping related sets. With the default parameters, the degree of AB-EF combined-impossibility would thus be, two plus one, three.
